# The complete mitochondrial genome of record-breaking migrant Arctic tern *(Sterna paradisaea)*

**DOI:** 10.1080/23802359.2019.1644225

**Published:** 2019-07-22

**Authors:** Ilze Skujina, Amy Jane Elizabeth Healey, Sophie de Becquevort, Paul William Shaw, Robert McMahon, Charly Morgan, Caron Evans, Rachel Taylor, Matthew Hegarty, Niall Joseph McKeown

**Affiliations:** a Institute of Biology, Environmental and Rural Sciences at Aberystwyth University, Aberystwyth, Wales, UK;; b Department of Molecular Haematology, Haematology Laboratory, Royal Infirmary of Edinburgh, Edinburgh, Scotland, UK;; c British Trust for Ornithology, Thetford, UK

**Keywords:** *Sterna paradisaea*, mtDNA, complete mitogenome, long-lived migrant

## Abstract

The analysis of mitochondrial DNA (mtDNA) base composition, codon usage, and genome arrangement patterns can provide insight into metabolic pathways and evolutionary history. Here, we report on the complete mitochondrial genome (mitogenome) of Arctic tern (*Sterna paradisaea*) a species notable for undertaking the longest migrations of any species as well as breeding in sub-polar habitats and capable of enduring extreme altitude. The complete mitogenome was 16,708 bp long and was typical of other avian mitogenomes in size and content. The phylogenetic position of the Arctic tern within Charadriiformes based on the coding region on the mtDNA corresponded closely to that based on nuclear loci. The sequence will provide a useful resource for investigations of metabolic adaptations of this remarkable species.

The terns (*Charadriiformes*: *Laridae*: *Sternini*) are cosmopolitan core water birds closely related to the gulls, noddies, and skimmers. The life histories of 12 generally accepted genera of terns range from that of entirely sedentary to strongly migratory species (Cramp 1985). Among these, the Arctic tern (*Sterna paradisaea*) holds the record for the longest roundtrip animal migration ever recorded electronically (Egevang et al. 2010). Arctic terns also reach long-lifespans despite life-long high energy expenditures and exposure to hypoxia associated with extreme conditions such as cold and high altitude flight (Hatch 1974). We used Illumina MiSeq platform to sequence the whole genome from heart tissue of a casualty Arctic tern (N53.42129° W4.60853°) – kindly donated by British Trust for Ornithology (available at IBERS Natural History Museum Tissue Collection: accession AT001). Shotgun libraries were prepared using the *Nextera XT* DNA Sample Preparation Kit (Illumina, San Diego, USA) of *Qiagen DNeasy* (Qiagen Ltd, West Sussex, UK) extracted DNA from mitochondrial-enriched DNA fraction obtained by differential centrifugation. The *Illumina MiSeq* run generated 8,468,804 paired-end reads that were mapped to complete *Sterna hirundo* genome (NCBI GenBank: MF582632; Yang et al. 2017) used as a reference on *CLC Genomic Workbench* v3.6 (CLC Bio, Aarhus, Denmark). In total, 1.09% of the raw reads mapped to the *Sterna hirundo* mtDNA and yielded two ∼12kb and ∼3 kb contigs that covered 95.62% of the reference genome. Sanger sequencing was used, to add the missing control region (CR) sequence (1158 bp) and to confirm that no CR duplications (Skujina et al. 2017) was present in this species. The complete mitogenome sequence of the Arctic tern (GenBank: MK946458) was 16,708 bp with heavy strand GC composition of 44.10% and encoded the 37 vertebrate mtDNA genes in the order that is considered standard for the Aves (Gibb et al. 2007).

For 10 out of the 13 mitochondrial protein coding genes (PCG) of *Sterna paradisaea,* the traditional AUG codon was used for initiation (Drabkin and RajBhandary 1998), whereas COX1 and ND5 genes were initiated by GUG and ND3 by AUC. Traditional mitochondrial open reading frame stop codons of AGG, AGA, UAA, or UAG was used for all genes apart from COX3, ND2, and ND4 which had incomplete stop codon (T) that is completed by the addition of 3′ A residues to the mRNA. The ND3 gene had a nucleotide base C in 9693 site that is not translated, although the frameshift mechanism is unknown (Mindell et al. 1998).

Phylogenetic position of the newly assembled complete Arctic tern mitogenome coding sequence within the Charadriiformes was resolved in MEGA 7 (Kumar et al. 2016) by maximum likelihood (ML) tree (Figure 1) following the method of Tamura and Nei (1993). Phylogenetic relationships aligned with those reported by Prum et al. (2015) based on 259 nuclear loci including 390,000 bp of 198 species of extant birds. Given its central role in energy metabolism, comparison of the mitochondrial DNA (mtDNA) of closely related species with different life-histories should provide a better understanding of the adaptive response to environmental stress.

**Figure 1.**
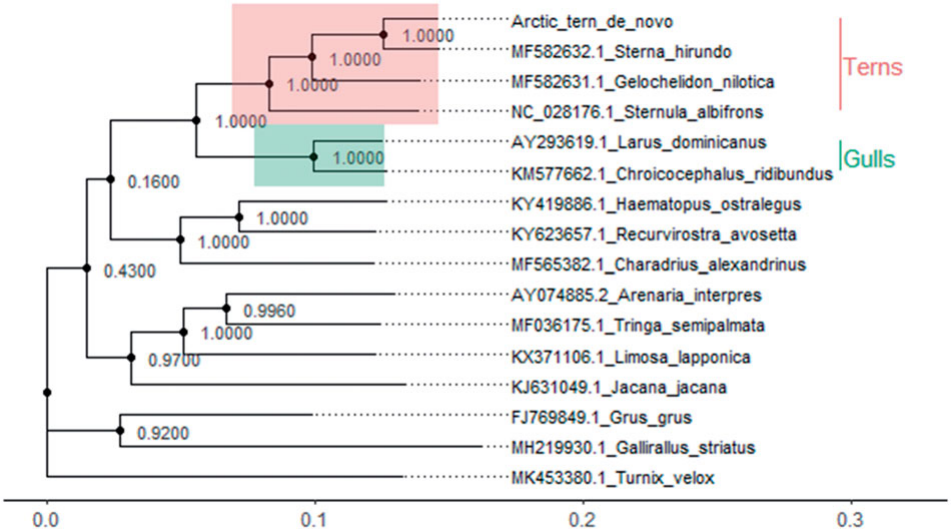
ML inferred phylogenetic relationships among *Charadriiformes* based on available mitochondrial coding region nucleotide sequences that corresponded to high resolution avian nuclear phylogenetic tree as constructed by (Prum et al. 2015). Annotation was created in R using ‘ggtree’ package (Yu et al. 2017, 2018).
